# Identification of the health education targeted susceptible population of tuberculosis in Ningxia, Northwest China

**DOI:** 10.1038/s41598-024-63961-5

**Published:** 2024-06-06

**Authors:** Ning Ma, Linlin Chen, Fan Ding, Xianglong Liu, Jiangping Li, Yu Zhao

**Affiliations:** School of Public Health, Ningxia Medical University, Ningxia, China

**Keywords:** Tuberculosis, KAP, Latent classification, Mediation effect, Susceptible population, Health care, Risk factors

## Abstract

Knowledge, Attitude, and Practice (KAP) survey, as an effective measure tool, is of practical significance for identifying the susceptible population in high-incidence regions of tuberculosis (TB). We aim to identify the health education targeted susceptible population of TB and discuss the acting pathway of KAP in Ningxia. A multistage random sampling method was used to conduct a face-to-face questionnaire survey for residents. The latent class analysis (LCA) model was used to classify susceptible populations of TB, and the structural equation modeling (SEM) model was also employed to investigate the interaction mechanisms of KAP (mediation analysis). We further applied the ordered logistic regression model to explore the associated factors. A total of 973 residents were enrolled, 70.6% were male, aged from 16 to 89. The LCA analysis demonstrated that 3 categories of susceptible populations of TB ("overall good", "positive attitude" and "overall poor") have optimal goodness of fit (BIC = 7889.5, Entropy = 0.923). SEM model indicated that the attitude plays a significant mediation effect from knowledge to practice toward TB (an indirect effect of 0.038, and a direct effect of 0.138). The ordered logistic regression results found that age, sex, marital status, education level, occupation, family income, self-perceived health status, having a family member or friend with TB, and knowing the DOTS strategy were significantly associated with classifications of KAP level towards TB. Based on the LCA model, we accurately classified the susceptible population of TB into 3 groups with different degrees of KAP. We found that TB attitude plays a mediating role between knowledge and practice. Therefore, we should pay more attention and carry out targeted health education in the community to these populations with overall poor KAP towards TB, and develop effective strategies and measures to realize the End TB Plan.

## Introduction

As an infectious disease that has existed for thousands of years, tuberculosis (TB) remains one of the leading causes of death globally. Early prevention and intervention of TB have received sustained attention and urgent action globally. The World Health Organization (WHO) reports that the COVID-19 pandemic has a severe impact on tuberculosis, leading to an increase in the number of undiagnosed and untreated tuberculosis patients, an increase in the incidence of community-acquired infections, and consequently the potential for a rise in the number of deaths^1^. The serious consequences of this damaging impact have undoubtedly altered the effective progress achieved in TB prevention and control. Although the country has been giving high priority to the prevention and treatment of TB, China still added 780,000 new TB patients, with a fatality rate of 4% (95% CI 3% ~ 5%) in 2021^2^. The reported incidence of TB in Beijing is 30.68 per 100 000, while in Ningxia the incidence is 38.68 per 100 000 in 2021^3,4^. Thus, it is urgent to strengthen the efficiency of prevention and control against TB to realize the End TB Plan on time.

Health education, as a major intervention strategy for TB control, may be beneficial to guide the correct behaviors of the population towards prevention and treatment of TB^5–7^. The health behavior change was significantly associated with the incidence of TB and contributes to improving adherence to the quality of prevention and control of TB^8,9^. Health behavior change toward TB is a complex decision-making process, which is influenced by socioeconomic, socio-recognition factors, and individuals’ willingness at multiple levels^10^. Knowledge, Attitude, and Practice (KAP) survey, as an effective measurement tool, is of significance for understanding the current level and deficiencies of health behavior change towards TB^7^. In addition, the adoption of correct TB practices is driven by intrinsic factors, such as perception, attitude, knowledge, etc.^11^. The acting path of these intrinsic factors may influence the outcomes of TB health behavior practices. Thus, the interaction mechanism of KAP may be conducive to deeply understanding the key process of health behavior change of TB. Given this, the implementation of targeted health education to the susceptible population of TB with different characteristics may improve the effectiveness of health promotion of TB, it is therefore necessary to classify the susceptible population of TB based on the KAP survey, and then carry out more accurate targeted health education based on the acting path of KAP to improve health behavior towards TB.

Generally, the KAP of individuals is difficult to observe directly (generally viewed as latent variables). Recently, structural equation modeling (SEM) and latent class analysis (LCA) have been widely used as practical techniques for dealing with latent variables in fields such as psychology and preventive medicine^12,13^. LCA explains associations between exogenous indicators through intermittent latent variables and constructs a model to achieve latent category clustering^14,15^. SEM not only evaluates measurement error but also assesses the direct and indirect effects of variables^16,17^. Therefore, we used the LCA model to classify the susceptible population of TB in Northwest China based on KAP, and then we also employed the SEM model to investigate the interaction mechanisms of KAP (mediation analysis). These results may provide a reliable reference for the development of effective intervention policies and measures for TB.

## Materials and methods

### Study population and sampling method

As an economically underdeveloped region with a significant proportion of the agricultural population(about 33.66%), Ningxia may face a higher incidence and heavier disease burden of TB^18^. Thus, this study adopts a multistage random sampling method to carry out the KAP survey on TB in Ningxia from 2022. In the first stage, 13 towns and streets were randomly selected in Guyuan City based on PPS (Probability Proportional to Population Size). In the second stage, 52 communities and villages were chosen from the 13 towns and streets level using the PPS method. In the second stage, residents aged 16 years and above were randomly selected from each village or community for face-to-face questionnaire surveys using the simple random sampling method. Several studies focusing on the age group of 16 may help to understand the effects of education policies and practices^19,20^. The sample covered a population with diverse characteristics such as varying ages, genders, educational backgrounds, and income levels, demonstrating good representativeness.

**Figure 1:**
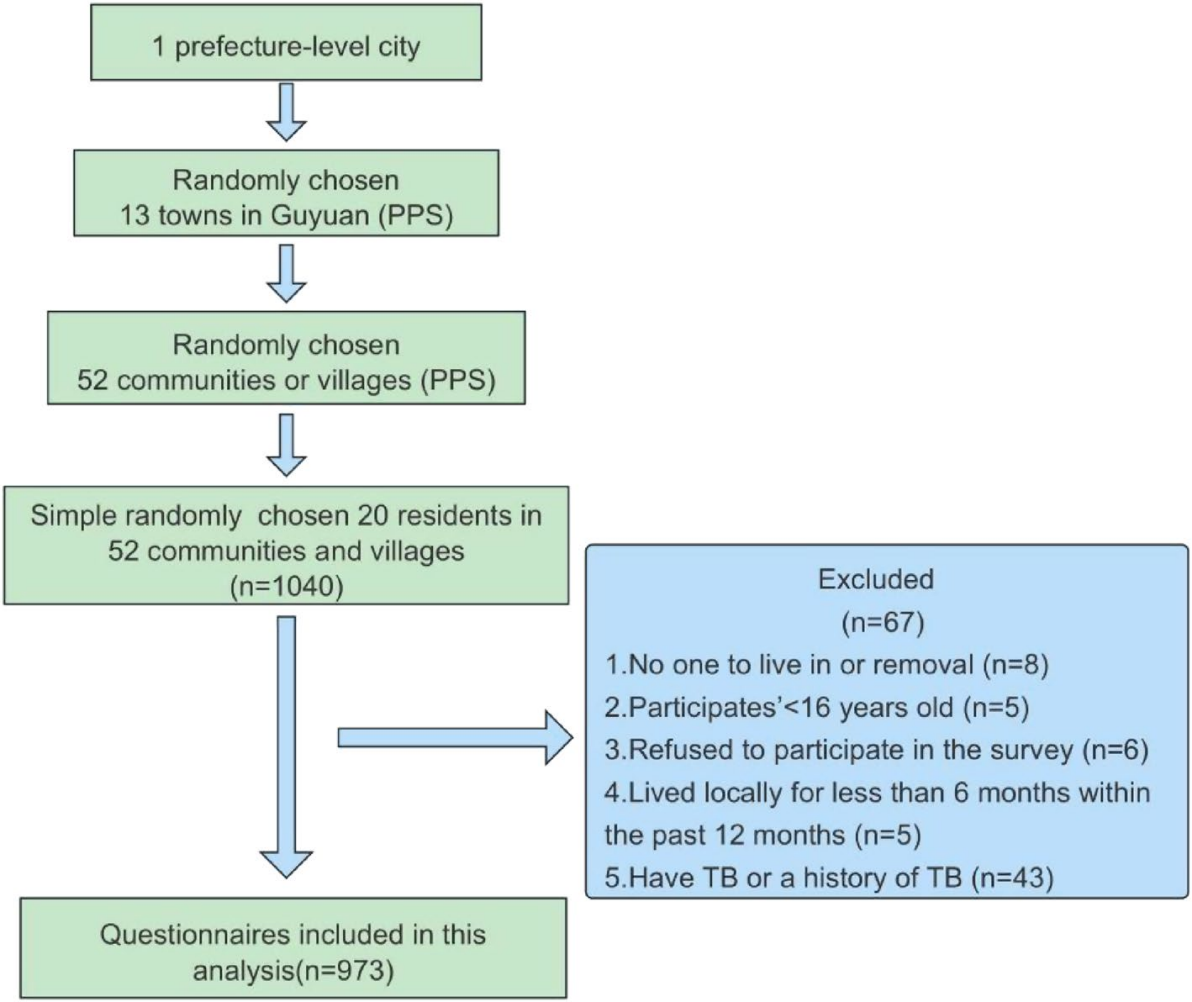
The study flowchart.

Inclusion exclusion criteria: (1) lived locally for more than six months within the past 12 months; (2) willingness to participate in this survey; (3) aged 16 years and older; (4) had basic word reading and communication skills; (5) no serious mental illness affecting the investigation; (6) no TB or a history of TB. Those who met the above requirements were included in the investigation, and those who did not meet were excluded. A total of 973 subjects eventually were included and the refusal rate of this survey was 0.58%. (For more details see Fig. 1). The sample size was calculated by the formula^21^:

1 $$N=deff\cdot \frac{{\mu }^{2}p(1-p)}{{d}^{2}}$$

where *p* represents the total awareness rate, based on the overall awareness rate regarding the knowledge of TB prevention and control in Ningxia, we set *p* = 0.724^22^, the relative error *r* = 5%, the critical value of confidence *u* = 1.96, the efficiency value *deff* = 1.5, and absolute error *d* = 5%*72.4% = 3.62%. The sample size was calculated to be about 880. We also assumed that a non-response rate of 10% and a final sample size is approximately 970.

#### Ethical permission

All respondents participating in the questionnaire survey before the survey have their own or guardian's consent and sign the informed consent. This study was approved by the ethics committee of the review board of Ningxia Medical University (Ethics Committee of Ningxia Medical University, No. 2020–095). Research on all research participants was performed in accordance with the Declaration of Helsinki. This study was performed in accordance with all relevant guidelines and regulations.

### Questionnaire design and survey contents

The questionnaire was developed based on the World Health Organization's "Advocacy, communication and social mobilization for TB control: a guide to developing knowledge, attitude and practice surveys^23^," as well as the "2006 National Tuberculosis Control Public Knowledge, Attitude, and Practice Survey Questionnaire" of China. It was modified through two rounds of surveys to adapt to the specific circumstances of the study area^24^.

The questionnaire includes (1) demographic characteristics: sex, age, marital status, education level, occupation, family income, self-assessed health status, basic medical insurance, family members or friends having TB, know Directly-Observed Treatment Strategy (DOTS); (2) TB knowledge; (3) TB attitude; (4) TB practice. The knowledge, attitude, and practice of TB include three aspects. (See in Table 1 for more details).

**Table 1:** The characteristics of study populations.

	Items	Score
Knowledge	Symptoms of TB	1 = Yes, 0 = No
	Route of transmission of TB	1 = Yes, 0 = No
	How to prevent TB	1 = Yes, 0 = No
Attitude	Whether willing to know information about TB	1 = Yes, 0 = No
	Whether willing to participate in TB health education activities	1 = Yes, 0 = No
	Whether willing to complete treatment of TB	1 = Yes, 0 = No
Practice	Actively understand the information about TB	1 = Yes, 0 = No
	Whether the mouth and nose are covered when coughing or sneezing	1 = Yes, 0 = No
	If you have TB, whether insist on treatment	1 = Yes, 0 = No

### Quality control

Investigators and data entry personnel received uniform and rigorous training. The survey was conducted face-to-face. After each day’s survey, the investigators checked the questionnaires and supplemented any missing or incorrect information in time. EpiData3.02 software was used for double entry to ensure accuracy, logical proofreading, and consistency checks.

### Statistical analysis

#### Latent class analysis

LCA is a statistical method for parameter estimation based on the response patterns of individuals on the observed indicators, that is, different joint probabilities. In LCA, which applies to categorical observed data, the observed patterns are presumed to be “caused” by each observation's relationship to an unmeasured variable. The value of this unmeasured variable is the latent classification and may consist of two or more actual classes^25^.

In the questionnaire, a value of 1 was assigned to the option "Yes," and a value of 0 was assigned to the option "No" for single-choice questions. For multiple-choice questions, a score of 1 was given if 60% or more options were correctly selected, while a score of 0 was given if the correct options were unknown or less than 60%. Each item was a binary variable in the inclusion of potential category analysis. The optimal LCA model was selected by the fit indices included: (1) information criterion indices-Alaike’s information criterion (AIC), Bayesian information criterion (BIC); (2) the bootstrap likelihood ratio test (BLRT). For these fit indices, simulation studies have demonstrated that BIC and BLRT are the best^26^. The lower the value of BIC represents a better fitness of the model. Entropy with a score above 0.8 is considered to be of high quality^27^. If the optimal LCA model is fitted, the latent class attributes of each need to be determined. In LCA, the potential category to which the individual of the combination should belong is determined based on Bayesian Posterior Probability, which serves as the classification standard^28^.

#### Mediation effect analysis

The previous study analyzed the direct effect of knowledge and attitude on practice towards TB, but the specific impact mechanism based on latent variables for this has not yet been studied^29^. As a method for estimating and testing causal models, SEM has the ability to deal with latent variables, measurement errors, and causal relationships. The model consists of two parts: the measurement model which describes the relationship between latent variables and indicators, and the structural model which describes the relationship between latent variables^30^. Thus, we employ SEM to evaluate the direct and indirect effect of knowledge and attitude on practice towards TB.

#### Univariate ordinal logistic regression

Since we used the latent variables in the KAP survey to classify the TB susceptible, the individual will be divided into several classes according to their KAP levels. Given the demographic influencing factors of KAP classification for TB susceptibility remain unclear, we utilized the KAP classification as a ranked response variable and performed a univariate ordinal logistic regression model to explore the potential influencing factors of TB susceptibility classification. More details for the variable assignment Table were given in Supplement 1.

#### Sensitivity analysis

Due to some of the demographics of the survey data being imbalanced, it is necessary to perform the weighted logistic regression to validate the robustness and consistency of our main results^31,32^. Considering the imbalance in data of sex and age, we have defined the weights corresponding to sex and age respectively, more details are in Supplement 2.

#### Statistical software

Mplus 8.3 was used to estimate the LCA model and construct the structural equation model. SPSS 23.0 was used for basic statistical analysis. The graphs were produced by using R packages "gcookbook", "ggplot2", and "Rmisc" in R 4.2.1.

### Ethics statement

The study was approved by the Ethics Committee of the Ningxia Medical University Institutional Review Board, Yinchuan, China (Ethics Committee of Ningxia Medical University, No. 2020–095). Informed consent was obtained from all survey participants. Research on all research participants was performed in accordance with the Declaration of Helsinki. This study was performed in accordance with all relevant guidelines and regulations.

## Result

### Demographic characteristics

Among 973 participants, 687 (70.6%) were male. Most people had a primary school education or lower (69.0%) and junior high school or senior high school education (27.4%). 69.5% of residents had an annual family income of less than 30,000 RMB. (For more details see Table 2).

**Table 2:** The characteristics of study populations.

Variables		N	%
Age	16–39	197	30.5
	40–59	479	49.2
	≥ 60	297	20.2
Annual family income (RMB)	< 20,000	299	30.7
	20,000–30,000	378	38.8
	> 30,000	296	30.4
Sex	Male	687	70.6
	Female	286	29.4
Marital status	Unmarried	76	7.8
	Married	825	84.8
	Divorce/widowed	72	7.4
Education level	Illiteracy/Primary school	671	69.0
	Junior high school/Senior high school	267	27.4
	University or higher	35	3.6
Occupation	Administrators/Teachers/Medical workers	31	3.2
	Farmers	776	79.8
	Workers/Students/Others	166	17.1
Self-perceived health status	Very good	237	24.4
	Good	265	27.2
	Fair	289	29.7
	Poor	131	13.5
	Very poor	51	5.2
Medical insurance	Yes	958	98.5
	No	15	1.5
Whether to know the DOTS (Directly-Observed Treatment Strategy)	Yes	103	10.6
	No	870	89.4
Family members or friends with TB history	Yes	84	8.6
	No	889	91.4

### Classification of susceptible of TB

Models fitted results with 1 to 7 latent classes are displayed in Table 3. By comprehensively comprising the goodness of fit indices (BIC, Entropy), we found that the 3-class and 4-class models have almost the same fit performance with larger entropy and smaller BIC. However, taking account into the practicality and simplicity, we selected the 3-class model as the final result.

**Table 3:** Model results for different numbers of potential classes.

Model	Loglikelihood	Df	AIC	BIC	Entropy	BLRT *P* value
1-class	− 4905.3	9	9828.6	9872.5	–	–
2-class	− 4186.7	19	8411.4	8504.1	0.859	< 0.001
3-class	− 3845.0	29	7748.0	7889.5	0.923	< 0.001
4-class	− 3774.6	39	7627.2	7817.6	0.924	< 0.001
5-class	− 3727.4	49	7552.8	7791.9	0.916	< 0.001
6-class	− 3880.1	59	7531.7	7819.7	0.900	0.009
7-class	− 3688.9	69	7515.9	7852.7	0.907	< 0.001

**Figure 2:**
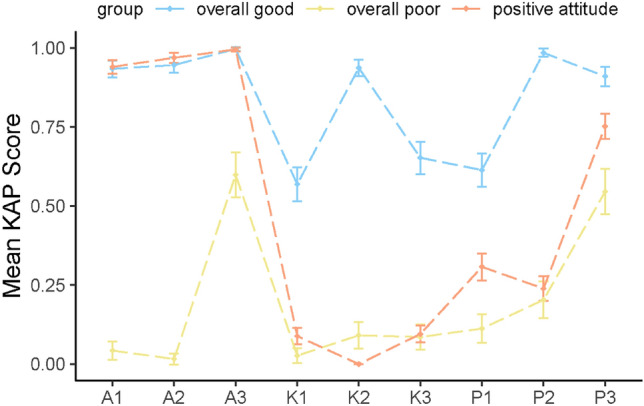
Mean KAP scores for 3 categories.

As demonstrated in Fig. 2, the average KAP scores of 3 classes (class 1–3) with different KAP levels of TB. The X-axis represents the latent variables (A1-A3, K1-K3, and P1-P3) used for the LCA analysis, and the Y-axis indicates the mean of the KAP scores. Error bars represent 95% of *CI* of average KAP scores.

Class-1 was labeled as “overall good (n = 334)” due to the high level of TB knowledge, attitude, and practice in this subgroup. Class-2 had the highest proportion of lack of TB knowledge but positive attitude and was labeled “positive attitude (n = 452)”. Class-3 was defined as “overall poor” (n = 187). It had generally poor TB knowledge, attitude, and practice. The knowledge of "symptoms of tuberculosis" of the three groups was low, and the “overall good” group was slightly higher than the other two groups.

### The acting pathway among knowledge, attitude and practice

**Figure 3:**
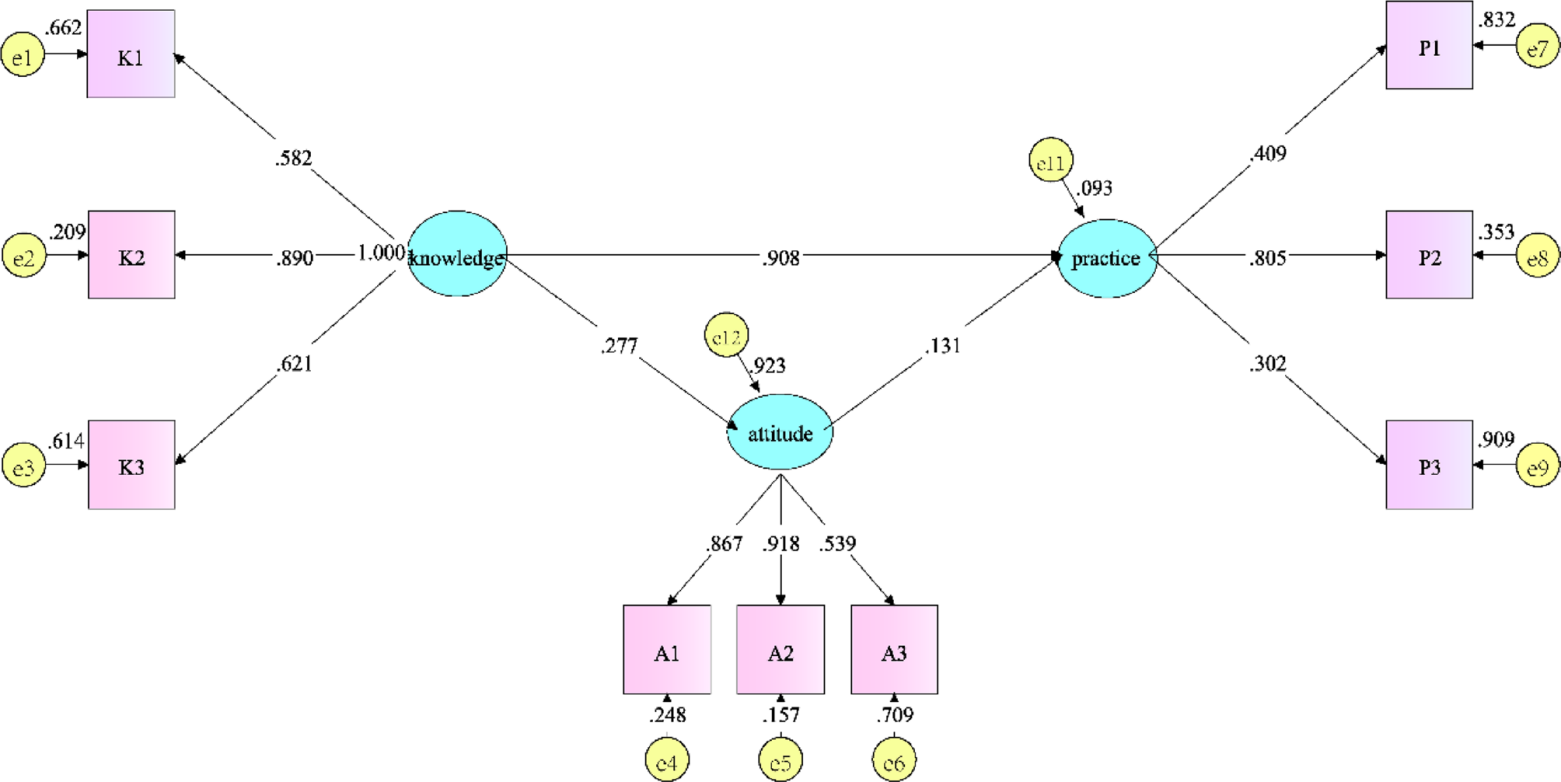
The final SEM all pathways were significant (*P* < 0.05).

Based on the LCA analysis, we categorized the susceptible population of TB into 3 classes, but the acting pathway among knowledge, attitude and practice needs to be further investigated. Accordingly, we used SEM analysis to discuss the direct and indirect impact of attitude on practice towards TB. According to the value of CFI, TLI, RMSEA and SRMR in Table. 4, with CFI = 0.931(> 0.90), RMSEA = 0.096(< 0.10) and SRMR = 0.058(< 0.08) all indicating that the goodness of fit measures of SEM meets the ideal standards. Therefore, the established SEM can be deemed acceptable. As shown in Fig. 3, the acting pathways among knowledge, attitude and practice consisted of two parts, the direct effect of knowledge on practice (standardized direct effects = 0.908), which demonstrated that the lower knowledge may induce a lower practice. The indirect effect of attitude between knowledge and practice (Standardized indirect effects = 0.036) (See Table 5 for more details). These results indicated that attitude plays a partial mediating role in the acting pathway from knowledge to practice towards TB.

**Table 4:** The fitted performance of structural equation model (SEM).

Fit	Statistics	Ideal standards
CFI	**0.931**	> 0.90
TLI	0.896	> 0.90
RMSEA	**0.096**	< 0.10
SRMR	**0.058**	< 0.08

Significant values are in bold.

*CFI* Comparative fit index, *TLI* Tucker Lewis index, *RMSEA* Root mean square error of approximation, *SRMR* Standardized Root Mean Square Residual.

**Table 5:** Bootstrap analysis of mediation effect significance test for the final model.

Acting pathways model	Standardized direct effects	*P* -value	Standardized indirect effects	*P* -value
Knowledge → practice	0.908	0.000	–	–
Knowledge → attitude	0.277	0.000	–	–
Attitude → practice	0.131	0.000	–	–
Knowledge → practice	–	–	0.036	0.000

### The influencing factors of TB susceptible population KAP classification

Table 6 presents the ordinal logistic regression results of the TB susceptible population’s KAP classification towards TB. The result revealed that male is more likely to have a better KAP level towards TB than female (*OR* = 1.34(1.04 ~ 1.74)). With the increase of age, the KAP level is decreased, that is, the middle-aged (*OR* = 0.41(0.29 ~ 0.58)) and elderly people (*OR* = 0.70(0.51 ~ 0.96)) possess a lower KAP classification. Compared with the married population, the divorced or widowed (*OR* = 0.56(0.35 ~ 0.88)) have a lower KAP level. With the education level enhanced, the KAP level towards TB significantly increased. It is worth noticing that farmers are the high-risk population of low KAP level (*OR* = 0.12(0.05 ~ 0.29)) and lower family income (*OR* = 1.57(1.18 ~ 2.09); *OR* = 1.87(1.38 ~ 2.54)) was also a disadvantage to the increase of individuals’ KAP level. The KAP level is significantly positively associated with the self-perceived health status (ORs increase with the improvement of health status *OR* = 2.13(1.20 ~ 3.77)). In addition, we also found that family members or friends have a TB history (*OR* = 1.60(1.05 ~ 2.46)) and know the DOTS (*OR* = 8.32(5.14 ~ 13.48)) benefit from the enhancement of KAP level. Additionally, as displayed in Table. S2, the weighted logistic regression results of KAP classification towards TB after adjusting weights of sex (or age group) indicated that the main results (see Table. 6) were consistent with the weighted logistic regression results. Thus, our results were robust.

**Table 6:** The ordinal logistic regression of KAP classification towards TB under demographic differences.

Variables	*OR* (95% *CI*)	*P* value	Test of parallel lines
Sex (Ref: Female)	–	**0.025**	0.345
Male	**1.34 (1.04 ~ 1.74)**	0.026	–
Age (Ref:16–39)	–	** < 0.001**	0.417
40–59	**0.41 (0.29 ~ 0.58)**	< 0.001	–
≥ 60	**0.70 (0.51 ~ 0.96)**	0.025	–
Marital status (Ref: Married)	–	**0.006**	0.523
Unmarried	1.47 (0.94 ~ 2.30)	0.088	–
Divorce/widowed	**0.56 (0.35 ~ 0.88)**	0.011	–
Education level (Ref: Primary school and below )	–	** < 0.001**	0.269
Junior college or below	**9.23 (4.10 ~ 20.77)**	< 0.001	–
University and above	**2.17 (1.65 ~ 2.85)**	0.001	–
Occupation (Ref: Teacher/Medical/Administrators)	–	** < 0.001**	0.193
Farmers	**0.12 (0.05 ~ 0.29)**	< 0.001	–
Workers/Students/Others	0.12 (0.05 ~ 0.32)	0.688	–
Family income (Ref: < 20,000)	–	** < 0.001**	0.165
20,000–30,000	**1.57 (1.18 ~ 2.09)**	0.002	–
> 30,000	**1.87 (1.38 ~ 2.54)**	< 0.001	–
Self-perceived health status (Ref: Very poor)	–	** < 0.001**	0.679
Very good	**2.13 (1.20 ~ 3.77)**	0.010	–
Good	1.47 (0.84 ~ 2.58)	0.181	–
Fair	1.02 (0.58 ~ 1.78)	0.949	–
Poor	0.75 (0.41 ~ 1.38)	0.358	–
Family members or friends with TB history (Ref: No)	–	**0.026**	0.334
Yes	**1.60 (1.05 ~ 2.46)**	0.029	–
Medical insurance (Ref: No)	–	0.125	0.280
Yes	2.20 (0.84 ~ 5.73)	0.107	–
Know the DOTS (Ref: No)	–	** < 0.001**	0.626
Yes	**8.32 (5.14 ~ 13.48)**	< 0.001	–

Significant values are in bold.

## Discussion

In this study, LCA model based on the KAP survey provided a optimal classifications of susceptible population of TB, that is, overall better (34.3%), lack of knowledge but a positive attitude (46.8%), and overall poor (18.9%) of TB in Ningxia. Most existing studies generally simply summed up the scores of items in the questionnaire to classify the TB status of the population and to explore the factors affecting KAP^33–40^. However, the use of KAP questionnaires inevitably involves latent variables, especially the KAP of the population, which may lead to underestimation or overestimation of the individual's level of health literacy. The classification of LCA is an extension of existing research on the KAP survey towards TB.

SEM analysis identified the mediating mechanism of KAP towards TB. More precisely, the direct effect of knowledge on attitude is 0.277 (*P* < 0.05), indicating that knowledge has a significant influence on attitude. The direct effect of attitude on practice is 0.131 (*P* < 0.05), and the direct effect of knowledge on practice is 0.908 (*P* < 0.05), indicating that both knowledge and attitude have a significant influence on behavior, and the influence of knowledge is stronger. Further results show that the indirect effect of knowledge on behavior through attitude is also significant (Standardized indirect effects = 0.036, *P* < 0.05). Attitude can be considered as a mediating variable between knowledge and practice towards TB. There is a close relationship between the knowledge, attitude and behavior towards TB. Knowledge, attitude and behavior affected each other and formed a dynamic and continuous process. There were different influencing factors among all dimensions. So it should not ignore the process of shaping health behaviors.

The acting pathways analysis among knowledge, attitude, and practice demonstrated that knowledge is the basis for practice change and attitude is the driver of practice change^41^. Both knowledge and attitude have a positive effect on practice. Adequate knowledge about the disease is necessary for good practice. If knowledge is inadequate, the probability of taking preventive measures will be low^42^. Similarly, there is a stronger relationship between attitude and practice, with a better attitude leading to more positive practice^43^. It has been shown that knowledge indirectly influences practice through attitude, however, the acting pathways for other diseases may differ from TB among knowledge, attitude, and practice^29^. It also can be seen that improving the TB knowledge of susceptible people may not only improve the attitude level but promote healthy behaviors as well. Due to the attitude only playing a partial mediating role in the relationship between knowledge and practice, simply improving the knowledge levels may not sufficient to promote healthy behavior formation. However, changing attitudes is a longer process, thus, introducing more examples of benefiting from behavioral changes may lead to attitude changes. Which also promotes behavioral change under the combined effect of knowledge and attitude^44^. These acting pathways results suggested that achieving positive attitude change among community residents can help in the design and implementation of strategic public health initiatives that may be beneficial to TB control.

The ordinal logistic regression indicated that the population with a “positive attitude” and “overall poor” had lower culture and economic levels compared with “overall good” for KAP towards TB, it was consistent with previous studies^45,46^. Residents with poor economic conditions and low education level often have lower integration into mainstream society, resulting in limited access to relevant knowledge and policies, thereby affecting their behaviors related to tuberculosis prevention and control^47^. Thus, we should appropriately focus health education and interventions for TB on those with lower literacy levels and family incomes. Age has a substantial impact on the population’s KAP level of TB, presenting a decrease in the level of knowledge, attitude, and practice for TB control with increasing age^38^. Compared to those with a high education level (such as medical personnel and teachers), farmers, laborers, and other professionals have considerable deficiencies in KAP levels. People with very poor self-perceived health have low KAP levels and may be more likely to be potentially susceptible to TB. Having a family member or friend who had TB was significantly associated with residents' KAP levels. Exchanging information may encourage individuals to learn about disease prevention timely^48^. Directly-Observed Treatment Strategy (DOTS) is one of the most cost-effective control strategies for TB that terminates TB infection and ensures effective treatment^49^. We found that people who understand the DOTS strategy were more likely to be categorized into the “overall good” classification. Popularizing the DOTS strategy can help us to improve the health behaviors of susceptible populations of TB, and increase the effectiveness of health promotion efforts^50^.

Conducting community health education can improve community residents' TB health literacy and positive health behaviors^51^. More interventions to increase KAP towards TB are needed to improve the prevention and control efficiency^52^. For instance, the dissemination of knowledge through digital platforms, SMS text messaging and phone call reminders, video call monitoring, and online lectures have raised the effectiveness of self-management, treatment adherence and prevention of TB in clinical and community settings^9,53,54^. Additionally, face-to-face PowerPoint slides and poster learning, focused discussion and answering questions with awards have improved residents' knowledge, attitudes and behaviors related to TB^55,56^. Thus, such targeted health education may greatly enhance the awareness of the population about TB prevention, thereby further guiding better attitudes and behaviors^57,58^.

This study still has some limitations. First, the sample population was drawn from the Ningxia region in Northwest China and is cross-sectional, the representation of the sample and the associations between variables are not as strong as the longitudinal data^59^. Second, the 3 classifications of KAP towards TB based on the LCA model have little overlap, which may be due to the fact that the residents' responses on the attitudinal and behavioral dimensions are too similar to be significantly different. Thus, larger sample populations should be collected in the future. Third, we only considered demographically related variables in the analysis of influencing factors and lacked the influence of environmental and psychological dimensions. It is of significance to further investigate the other characteristic variables and identify the susceptible population of tuberculosis more comprehensively. Fourth, during the survey period, Ningxia had strict control of COVID-19 and was in a low epidemic prevalence^60^, the mobility of residents was also limited, the COVID-19 pandemic may affect public awareness of infectious diseases^61^. However, all participants had homogeneous exposure levels, and the questionnaire items we designed were not directly related to COVID-19, resulting in our results being affected by the COVID-19 pandemic.

## Conclusion

Based on the LCA model, we accurately classified the susceptible population of TB into 3 groups with different degrees of KAP. We found that TB attitude plays a mediating role between knowledge and practice. The ordered logistic regression results found that age, sex, marital status, education level, occupation, family income, self-perceived health status, having a family member or friend with TB, and knowing the DOTS strategy were significantly associated with classifications of KAP level towards TB. Therefore, we should pay more attention and carry out targeted health education in the community to these populations with overall poor KAP towards TB, and develop effective strategies and measures to realize the End TB Plan on time.

### Supplementary Information

Supplementary Information.

## Data Availability

Data cannot be shared publicly because of the privacy of the participants. Data may be made available by contacting the corresponding author.

## Abbreviations

TB: Tuberculosis
KAP: Knowledge, attitude, practice
NHAR: Ningxia Hui Autonomous Region
DOTS: Directly-Observed treatment strategy
WHO: World Health Organization
LCA: Latent class analysis
SEM: Structural equation modelling
LCM: Latent class model
AIC: Alaike’s information criterion
BIC: Bayesian information criterion
BLRT: Bootstrap likelihood ratio test
