# Supplementary material for: Identification of the health education targeted susceptible population of tuberculosis in Ningxia, Northwest China
Source: Sci Rep. 2024 Jun 6;14:13071. doi: 10.1038/s41598-024-63961-5 (PMC11156889; doi:10.1038/s41598-024-63961-5)
Supplement: Supplementary file 1 — Supplementary Information. [file 41598_2024_63961_MOESM1_ESM.docx]

Table. S1 The variable assignment table

| **Variables** | **Types** | **Assignment rules** |
| --- | --- | --- |
| Class | Dependent variable | 1 = Overall poor, 2 = Positive attitude, 3 = Overall good |
| Age | Independent variables | 1 = ≥ 60, 2 = 40-59, 3 = 16-39 |
| Annual family income (RMB) | Independent variables | 1 = <20000, 2 = 20000-30000, 3 = >30000 |
| Sex | Independent variables | 1 = Male, 2 = Female |
| Marital status | Independent variables | 1 = Unmarried, 2 = Divorce/widowed, 3 = Married |
| Education level | Independent variables | 1 = University or higher, 2 = Junior high school/Senior high school, 3 = Illiteracy/Primary school |
| Occupation | Independent variables | 1 = Farmers, 2 = Workers/Students/Others, 3 = Administrators/Teacher/Medical workers |
| Self-perceived health status | Independent variables | 1 = Very good, 2 = Good, 3 = Fair, 4 = Poor, 5 = Very poor |
| Medical insurance | Independent variables | 1 = Yes, 2 = No |
| Family members or friends with TB history | Independent variables | 1 = Yes, 2 = No |
| Whether to know the DOTS  (Directly-Observed Treatment Strategy) | Independent variables | 1 = Yes, 2 = No |

**S2 Appendix. Sensitivity analysis**

Considering the imbalanced data of sex and age, we defined the following weighted formula$w_{i}$for adjusting sex:

$w_{i}=\frac{1}{x_{i}\frac{p_{s}}{P_{n}}+(1-x_{i})(\frac{1-p_{s}}{1-P_{n}})},$ (A.1)

here $x_{i}$ denotes the variables values of sex (=0 is female and =1 is male) for individual i, $p_{s}$is the proportion of sex (or age group) in survey and $p_{n}$ is the proportion of sex in total Ningxia. And we define the following weighted formula$w'_{j}$for adjusting age:

$w'_{ij}=\frac{1}{\frac{p_{ij}}{P_{jn}}},$ *j*=1,2,3, (A.2)

here $p_{ij}$ denotes the proportion of age group (*j*=1 is aged 16-39, *j*=2 is aged from 40-59, and *j*=3 is aged over 60) in this survey for individual *i*, $p_{jn}$is the proportion of *j*-th age group in total Ningxia.

Table. S2 The weighted logistic regression results of KAP classification towards TB after adjusting weights of sex or age.

| **Variables** | **Adjusting weights of sex** | | | **Adjusting weights of age** | | |
| --- | --- | --- | --- | --- | --- | --- |
|  | ***OR* (95%*CI*)** | ***P* value** | **Test of parallel lines** | ***OR* (95%*CI*)** | ***P* value** | **Test of parallel lines** |
| Sex (Ref: Female) | - | **0.013** | 0.303 | - | 0.096 | 0.335 |
| Male | **1.35 (1.06 ~ 1.71)** | 0.014 | - | 1.25 (0.96 ~ 1.62) | 0.097 | - |
| Age (Ref:16-39 ) | - | **<0.001** | 0.246 | - | **<0.001** | 0.463 |
| 40-59 | **0.37 (0.26 ~ 0.52)** | <0.001 | - | **0.70 (0.52 ~ 0.94)** | 0.016 | - |
| ≥60 | **0.65 (0.48 ~ 0.87)** | 0.005 | - | **0.41 (0.30 ~ 0.57)** | <0.001 | - |
| Marital status (Ref: Married) | - | **0.003** | 0.320 | - | **0.002** | 0.429 |
| Unmarried | **0.58 (0.38 ~ 0.88)** | 0.011 | - | **1.50 (1.00 ~ 2.26)** | 0.05 | - |
| Divorce/widowed | **1.51 (0.98 ~ 2.33)** | 0.006 | - | **0.52 (0.32 ~ 0.85)** | 0.008 | - |
| Education level (Ref: Primary school and below ) | - | **<0.001** | 0.198 | - | **<0.001** | 0.157 |
| Junior college or below | **7.63 (3.53 ~ 16.48)** | <0.001 | - | **2.15 (1.64 ~ 2.82)** | <0.001 | - |
| University and above | **2.40 (1.81 ~ 3.17)** | <0.001 | - | **7.89 (3.86 ~ 16.09)** | <0.001 | - |
| Occupation (Ref: Teacher/Medical/Administrators) | - | **<0.001** | 0.219 | - | **<0.001** | 0.287 |
| Farmers | **0.09 (0.03 ~ 0.25)** | <0.001 | - | **0.10 (0.04 ~ 0.26)** | <0.001 | - |
| Workers/Students/Others | **0.10 (0.03 ~ 0.29)** | <0.001 | - | **0.12 (0.05 ~ 0.32)** | <0.001 | - |
| Family income (Ref: <20,000) | - | **<0.001** | 0.396 | - | **<0.001** | 0.265 |
| 20000-30000 | **1.65 (1.23 ~ 2.20)** | <0.001 | - | **1.53 (1.13 ~ 2.06)** | 0.008 | - |
| >20000 | **2.05 (1.51 ~ 2.77)** | 0.001 | - | **1.92 (1.40 ~ 2.63)** | <0.001 | - |
| Self-perceived health status (Ref: Very poor) | - | **<0.001** | 0.488 | - | **<0.001** | 0.520 |
| Very good | **2.36 (1.30 ~ 4.26)** | 0.005 | - | **2.15 (1.17 ~ 3.97)** | 0.014 | - |
| Good | 1.43 (0.80 ~ 2.58) | 0.226 | - | 1.48 (0.81 ~ 2.71) | 0.205 | - |
| Fair | 0.95 (0.53 ~ 1.70) | 0.872 | - | 1.07 (0.58 ~ 1.96) | 0.837 | - |
| Poor | 0.70 (0.38 ~ 1.30) | 0.256 | - | 0.77 (0.40 ~ 1.49) | 0.443 | - |
| Family members or friends with TB history (Ref: No) | - | **0.005** | 0.168 | - | **0.020** | 0.482 |
| Yes | **1.81 (1.18 ~ 2.77)** | 0.006 | - | **1.68 (1.08 ~ 2.63)** | 0.022 | - |
| Medical insurance (Ref: No) | - | 0.590 | 0.210 | - | 0.252 | 0.230 |
| Yes | 1.30 (0.53 ~ 3.19) | 0.570 | - | 1.76 (0.70 ~ 4.42) | 0.570 | - |
| Know the DOTS (Ref: No) | - | **<0.001** | 0.806 | - | **<0.001** | 0.407 |
| Yes | **8.17 (5.06 ~ 13.18)** | <0.001 | - | **7.06 (4.42 ~ 11.27)** | <0.001 | - |

**Knowledge, attitude and behavior of tuberculosis among Ningxia resident’s questionnaire**

Hello! We are researchers at the School of Public Health, Ningxia Medical University, and we are conducting a study to understand residents' knowledge, attitudes, and behavior related to tuberculosis. The survey results are an important basis for evaluating the TB status of the city's residents and for providing TB health education and related policies.

Thank you for your support and cooperation!

Investigator： Survey Date：

**1. Basic information**

结**A01.** Sex**：**

① Male ② Female

**A02.** Year of birth**：**□□□□

结**A03.** Your Marital Status：

① Unmarried ② Married ③Divorce/widowed

结**A04.** Your education level：

① Illiteracy/Primary school

② Junior high school/Senior high school ③ University or higher

结**A05.** Your occupation：

① Administrators/Teacher/Medical workers ② Farmers

③ Workers/Students/Others

结**A06.** In the past year, what was your family's annual income (RMB)？

①≦12000 ② 12000-20000 ③≧20000

结**A07.** In the past year, how do you think your health：

① Very good ② Good ③ Fair ④ Poor ⑤ Very poor

结**A08.** Whether you have medical insurance？

① Yes ② No

结**A09.** Do you have family members or friends with TB history？

① Yes ② No

结**A10.** Do you know about the DOTS strategy (Directly-Observed Treatment Strategy for TB patients)?

① Yes ② No

**2. Knowledge, Attitude and Practice of tuberculosis**

结**B01.** What are the symptoms of pulmonary tuberculosis? (Multiple choice available)

① Coughing or coughing for more than 2 weeks

② Fever or fever for more than 7 days ③ Chest pain or hemoptysis

④ Shortness of breath or persistent weakness ⑤ Do not know

结**B02.** What are the ways of transmission of tuberculosis? (Multiple choices available)

① Droplets from coughing or sneezing ② Eating together or sharing utensils

③ Touching items in public places ④ Do not know

结**B03.** How to prevent tuberculosis? (Multiple choices available)

① Cover nose and mouth by coughing or sneezing

② Wash hands after touching things

③ Ventilation in the home ④ Good nutrition ⑤ Do not know

结**C01.** Would you like to learn about tuberculosis?

① Yes ② No

结**C02.** Would you like to participate in TB health education activities?

① Yes ② No

结**C03.** If you had tuberculosis, would you be willing to complete treatment?

① Yes ② No

结**D01.** Have you ever volunteered for information about tuberculosis?

① Yes ② No

结**D02.** Do you cover your mouth and nose when you cough or sneeze?

① Yes ② No

结**D03.** If you have tuberculosis, will you adhere to treatment?

① Yes ② No
